# Equivalent glycemic load (EGL): a method for quantifying the glycemic responses elicited by low carbohydrate foods

**DOI:** 10.1186/1743-7075-3-33

**Published:** 2006-08-24

**Authors:** Thomas MS Wolever, Alison L Gibbs, Matt Spolar, Elinor V Hitchner, Colette Heimowitz

**Affiliations:** 1 Glycemic Index Laboratories, Inc., 36 Lombard Street, Suite 100, Toronto, Ontario, M5C 2X3, Canada; 2 Department of Statistics, University of Toronto, Toronto, Ontario, Canada; 3 Atkins Nutritionals, Inc., New York, NY, USA; 4 Cadbury Schweppes Science and Technology Center, Whippany, NJ, USA

## Abstract

**Background:**

Glycemic load (GL) is used to quantify the glycemic impact of high-carbohydrate (CHO) foods, but cannot be used for low-CHO foods. Therefore, we evaluated the accuracy of equivalent-glycemic-load (EGL), a measure of the glycemic impact of low-CHO foods defined as the amount of CHO from white-bread (WB) with the same glycemic impact as one serving of food.

**Methods:**

Several randomized, cross-over trials were performed by a contract research organization using overnight-fasted healthy subjects drawn from a pool of 63 recruited from the general population by newspaper advertisement. Incremental blood-glucose response area-under-the-curve (AUC) elicited by 0, 5, 10, 20, 35 and 50 g CHO portions of WB (WB-CHO) and 3, 5, 10 and 20 g glucose were measured. EGL values of the different doses of glucose and WB and 4 low-CHO foods were determined as: EGL = (F-B)/M, where F is AUC after food and B is y-intercept and M slope of the regression of AUC on grams WB-CHO. The dose-response curves of WB and glucose were used to derive an equation to estimate GL from EGL, and the resulting values compared to GL calculated from the glucose dose-response curve. The accuracy of EGL was assessed by comparing the GL (estimated from EGL) values of the 4 doses of oral-glucose with the amounts actually consumed.

**Results:**

Over 0–50 g WB-CHO (n = 10), the dose-response curve was non-linear, but over the range 0–20 g the curve was indistinguishable from linear, with AUC after 0, 5, 10 and 20 g WB-CHO, 10 ± 1, 28 ± 2, 58 ± 5 and 100 ± 6 mmol × min/L, differing significantly from each other (n = 48). The difference between GL values estimated from EGL and those calculated from the dose-response curve was 0 g (95% confidence-interval, ± 0.5 g). The difference between the GL values of the 4 doses of glucose estimated from EGL, and the amounts of glucose actually consumed was 0.2 g (95% confidence-interval, ± 1 g).

**Conclusion:**

EGL, a measure of the glycemic impact of low-carbohydrate foods, is valid across the range of 0–20 g CHO, accurate to within 1 g, and at least sensitive enough to detect a glycemic response equivalent to that produced by 3 g oral-glucose in 10 subjects.

## Background

Decreased postprandial glucose concentrations and diets with a low glycemic load (GL) are associated with reduced risk for cardiovascular disease [1,2], diabetes [3,4] and, perhaps, some forms of cancer [5,6]. In addition, generally, low carbohydrate [7-9] or low GL diets [10,11] result in greater weight loss than high carbohydrate diets over periods of 3–6 months and have a favorable effect on triglyceride and HDL cholesterol [12]. Food manufacturers have responded to the demand for low glycemic products by replacing sugars and starch in conventional foods with ingredients such as sugar alcohols, oligo- and polysaccharides, or glycerin. However, it is not known how to quantify the glycemic impact of low-carbohydrate foods accurately.

The GL of a given weight of food is the weight of glucose which would raise blood glucose by the same amount as that amount of food. If the portion of food contains g grams of available carbohydrate (avCHO), GL is defined as g × GI/100 [4,13], where GI is the glycemic index of the food. Recently it has been reported that the GL of various doses of 5 different foods, calculated from g and GI, does not differ greatly from the glycemic response elicited by GL grams of glucose [14]. However, only high carbohydrate foods were tested because, to determine GI, subjects need to be able to eat a portion of food containing at least 25 g avCHO [15]. Since portions of low-carbohydrate foods containing even 5–10 g avCHO may be too large for subjects to eat, it is not possible to determine their GI, and therefore, it is not possible to determine their GL.

We previously described a method of measuring the glycemic impact of low carbohydrate foods termed equivalent glycemic load (EGL) [16]. EGL is defined as the amount of avCHO from white bread which raises blood glucose to the same extent as one serving of the food. Thus, EGL is conceptually similar to GL except that the reference food is white bread instead of glucose. Unlike GL, EGL can be used to evaluate the glycemic impact of low-carbohydrate foods, containing 0 to 20 g avCHO; however, its validity has not been established.

Therefore, the purpose of this paper is to determine the validity of EGL. The first step was to determine the shape of the dose-response curve for the glycemic responses elicited by 0–20 g avCHO from white bread; this is necessary because the EGL calculation assumes a linear relationship. The approach taken to determine the accuracy of EGL was to compare the EGL values of small doses of oral glucose with the amounts actually consumed.

## Methods

The glycemic responses elicited by various test meals were measured in groups of healthy subjects drawn from a pool of 25 males and 38 females, aged 19–71 years with a body mass index of 18.5 to 36.5 kg/m^2^. The studies reported here were performed over a period of approximately 4 years. Each subject was studied on multiple occasions in the morning after 10–14 h overnight fasts using the same procedure each time. After a fasting blood sample had been obtained by finger-stick, subjects consumed a test meal within 10 min, and further finger-stick blood samples were obtained at 15, 30, 45, 60, 90 and 120 min after starting to eat. Test meals were consumed with a drink of the subject's choice consisting of 1 or 2 cups of water, coffee or tea with 30 ml 2% milk per cup if desired. The drink chosen by each subject remained the same for all tests in which the subject participated. Blood samples (2–3 drops) were collected into polypropylene tubes containing sodium fluoride and potassium oxalate and stored at -20°C prior to whole blood glucose analysis using an automatic analyzer (Model 2300 STAT, Yellow Springs Instruments, Madison, WI). Incremental areas under the blood glucose response curves (AUC), ignoring area below fasting, were calculated as previously described [17].

The shape of the dose response curve elicited by various doses of white bread was determined using all the tests of white bread performed by all subjects who participated in studies to determine the EGL of low-carbohydrate foods produced by the sponsor during the period between 2001 and 2004. Each subject participated in multiple tests which were grouped into blocks of 7 to 10 different test meals each. The first block done by each subject consisted of 4 doses of white bread (drink alone or a portion of white bread containing 5, 10 or 20 g avCHO, termed WB0, WB5, WB10 and WB20, respectively) in randomized order plus 4–6 foods (the results for the foods will be reported elsewhere). Every subsequent block of 7 tests usually consisted of 6 test foods and one randomly chosen dose of white bread, with all 4 doses of white bread being tested per 4 blocks. Thus, if a subject participated in 9 blocks, he or she would have tested each dose of white bread 3 times (each dose once in block 1, each dose once in blocks 2–5, and each dose once in blocks 6–9).

A total of 48 subjects (15 male, 33 female; 34 Caucasian, 6 East Asian, 1 Hispanic, 4 Middle Eastern, 2 South Asian, 1 African; age, 34.1 ± 1.9 y; BMI, 23.8 ± 0.6 kg/m^2^) consumed WB0, WB5, WB10 and WB20 at least once; there were 122 tests of WB0, 115 of WB5, 126 of WB10 and 123 of WB20. In addition 10 subjects took test meals consisting of portions of white bread containing 35 or 50 g available carbohydrate (WB35 and WB50), with 9 of the 10 subjects repeating each of these doses 2 times. White bread was baked in an automatic bread maker and cut into appropriate portion sizes as previously described [18].

**Figure 1:**
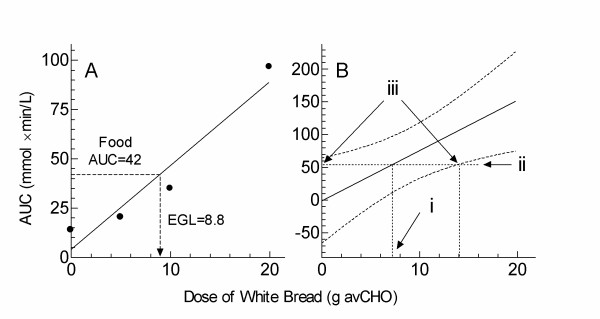
**Illustration of EGL calculations**. Points represent AUC values elicited by doses of white bread containing 0, 5, 10 and 20 g avCHO. A: Sample calculation of EGL using data from one subject. The regression equation (solid line) is: AUC = 4.3 g + 4.0 where g = grams of avCHO. Thus, if a serving of a test food elicits and AUC of 42, the EGL value is 8.8. B: Effect of uncertainty in estimate of the slope and y-intercept on EGL values. Data are from the subject whose data yielded a correlation coefficient equivalent, r = 0.971, equivalent to the average for all 20 subjects. The solid line is the regression equation and the curved dashed lines the 95% confidence band of the regression equation. For an EGL value of 7.1 (i), the corresponding AUC is 54 (ii); the 95% confidence interval for the EGL value corresponding to an AUC of 54 is 0–14 (iii).

The results of the glycemic responses elicited by different doses of white bread showed that, for doses of bread containing between 0 and 20 g avCHO, the AUC of blood glucose was a linear function of the amount of avCHO consumed as follows:

AUC = M × g + B     (1)

where g is the grams of avCHO from white bread, M is the slope and B is the y-intercept. For each subject, M and B were derived by linear regression analysis using AUC values elicited by WB0, WB5, WB10 and WB20. A sample calculation is shown in Figure 1A. Equation 1 can be rearranged to allow g to be calculated for any value of AUC as follows:

subtracting B from both sides: AUC - B = Mg

dividing both sides by M: (AUC - B)/M = g

Therefore: g = (AUC - B)/M     (2)

If we now consider a serving of a low carbohydrate food which elicits an AUC of AUC_f_, then the grams of avCHO from white bread which elicits a glycemic response equal to AUC_f _would be the EGL of that food. Thus, substituting in equation 2:

EGL = (AUC_f _- B)/M.     (3)

The EGL calculation was performed for each subject and the average taken to be the EGL of the food. If a subject's EGL was <0, the value was taken to be 0 on the grounds that it is not possible to have a negative AUC.

To determine the accuracy of EGL, 20 subjects (11 male, 9 female; 13 Caucasian, 3 East Asian, 2 Hispanic, 1 South Asian, 1 African; age, 37.7 ± 3.5 y; BMI, 25.2 ± 0.8 kg/m^2^) were studied on 18 different occasions. This series of tests, performed in 2005, were different from those described above. Each subject took 11 different test meals consisting of WB0, WB5, WB10 and WB20 (each dose of bread was done twice), 5 g, 10 g and 20 g glucose (G5, G10, G20; anhydrous glucose, Sigma Chemical Corp., St. Louis, MO), dissolved in 100 ml water (each dose of glucose was done twice) and one serving of each of 4 foods: Advantage Chocolate Delight Shake (1 can = 325 ml, 9 g fat, 20 g protein, 5 g total carbohydrate, 4 g dietary fiber), Advantage Chocolate Peanut Bar (1 bar = 60 g, 12 g fat, 19 g protein, 21 g total carbohydrate, 10 g dietary fiber), Endulge Caramel Nut Chew (1 bar = 35 g, 9 g fat, 6 g protein, 18 g total carbohydrate, 2 g dietary fiber) and Quick Quisine Pancake Mix (1 pancake = 10 g fat, 11 g protein, 9 g total carbohydrate, 3 g dietary fiber). The foods were chosen from those previously tested to cover a range of EGL values from about 1 g to 8 g. The EGL values for these 4 foods were calculated from the dose-response curves for white bread as described in equations (1) to (3) above.

EGL is calculated from the estimates of B and M from regression equation (1) above. To determine the effect of imprecision in the estimates of B and M on the resulting EGL value, EGL values for each of the 4 test foods described below were calculated from the extremes of the range bounded by the 95% confidence band of the regression line of AUC on dose of avCHO from white bread for the subject with a correlation coefficient (r) closest to the mean r value for all 20 subjects. The AUC expected for the EGL value of each food was determined from the regression line; for example the EGL of pancake mix was 7.1 g (i on Figure 1B) corresponding to an AUC of 54 mmol × min/L (ii on Figure 1b). A horizontal line at this AUC crosses the 95% confidence band at EGL values <0 g and ~14 g (iii on Figure 1B). Since EGL values <0 are taken to be 0, we would expect 95% of individual subject's EGL values for this food (pancake mix) to lie between 0 and 14 g. The expected range of individual EGL values for each food was compared with that observed in the 20 subjects.

If EGL was accurate, the value obtained for the small doses of glucose consumed should be equal to the amounts of glucose actually consumed, after adjusting for the difference in glycemic response between white bread and glucose. To determine how to estimate GL from EGL, we solved the simultaneous dose-response equations for the glycemic responses elicited by different doses of white bread and glucose as follows:

As shown in equation (1) above, the relation between AUC and dose of avCHO from white bread is described by the following equation:

AUC = M_wb _× g_wb _+ B_wb _    (4)

where M_wb _is the slope, B_wb _is the y-intercept and g_wb _is the grams of avCHO from white bread consumed. Equation (2) above shows how g_wb _can be calculated from AUC, and equation (3) shows that the g_wb _calculated from the glycemic response elicited by a food, AUC_f_, is equivalent to the EGL of the food. Therefore, equation (4) can be re-written as:

AUC_f _= M_wb _× EGL + B_wb _    (5)

Similarly, the relation and between AUC and dose of glucose is:

AUC = M_g _× g_g _+ B_g _    (6)

where M_g _is the slope, B_g _is the y-intercept and g_g _is the grams of glucose consumed. By analogy with equation 3, the values of M_g _and B_g _from equation (6) can be used to calculate the grams of glucose which elicits any given AUC, which is, by definition, equivalent to GL. Thus, if the AUC elicited by a serving of food is AUC_f_, its GL is calculated as follows:

GL = (AUC_f _- B_g_)/M_g _    (7).

Equation (7) can be re-written as:

AUC_f _= M_g _× GL + B_g _    (8).

If, in equations (5) and (8) above, the value for AUC_f _is the same, we can write:

M_wb _× EGL + B_wb _= M_g _× GL + B_g _    (9).

Solving equation (9) for GL yields the following:

GL = [(M_wb _× EGL) + (B_wb _- B_g_)]/M_g _    (10).

To test the accuracy of equation (10), the EGL of the 10 different test meals were calculated in each subject using the mean AUC for the 2 tests of each dose of white bread taken by the same subject. Similarly the GL values were calculated using the mean AUC for the 2 tests of each dose of glucose taken by each subject. The observed GL values were compared with those calculated from the EGL values using equation (10).

The accuracy of EGL was assessed using the EGL values for 5, 10 and 20 g glucose determined in the experiment described above. In addition, the EGL of 3 g glucose dissolved in 100 ml water was determined 2 times in one group of 10 subjects. None of the subjects who tested 3 g glucose participated in the experiment in which 5, 10 and 20 g of glucose were tested. The GL values for each of the 4 doses of glucose were estimated from EGL using equation (10). If EGL is accurate, the resulting GL values should be equal to the grams of glucose actually consumed.

Results are presented as means and SEM, unless otherwise noted. The influence of sex, ethnicity, age and BMI on AUC and EGL values were assessed by multiple linear regression analysis (Lotus 1–2–3 97 Edition, Lotus Development Corporation, Cambridge, MA) using dummy variables for sex (0 = female, 1 = male) and ethnicity (0 = Caucasian, 1 = Other). 95% confidence bands of regression models were fit using Prism 4 (GraphPad software, San Diego, CA). Comparison of linear and non-linear mixed models was performed on SAS (version 8.2, SAS Institute Inc., Cary, NC) using corrected Akaike's Information Criterion [19]. Repeated measures analysis of variance was used to examine for the significance of differences in AUC between different doses of white bead and glucose. After demonstration of significant heterogeneity, Tukey's test was used to test the significance of the differences between individual means with p < 0.05 (2-tailed) being used as the criterion for statistical significance. Expected and observed values were compared using the Bland-Altman analysis [20].

The protocol was approved by the Western International Review Board^® ^and all subjects gave their informed consent to participate by signing the approved consent form.

## Results

### Shape of dose-response curve for white bread

**Figure 2:**
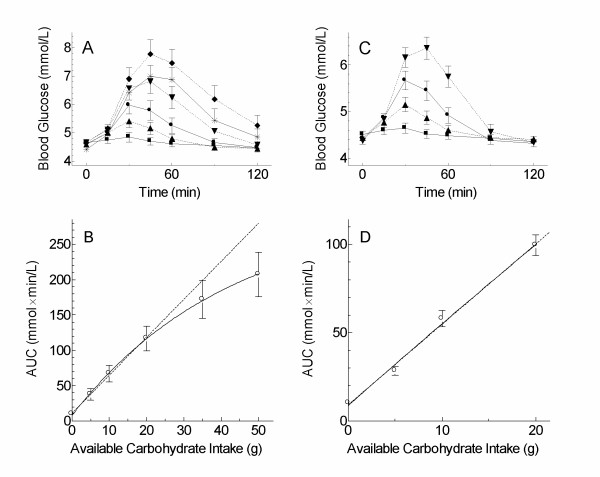
**Blood glucose responses elicited by different doses of white bread**. A: blood glucose concentrations in n = 10 subjects after 0 (■), 5 (▲), 10 (●), 20 (▼), 35 () and 50 g (◆) carbohydrate; B: incremental areas under the curve (AUC) for data in panel A, solid line is non-linear regression through all points (AUC = 291 × (1-e^-0.0233 g^) + 7.84), dashed line is linear regression through 0, 5, 10 and 20 g points (AUC = 5.38 g + 10.9); C: blood glucose concentrations in n = 48 subjects after 0, 5, 10 and 20 g carbohydrate; D: AUC values for data in panel C, solid line is non-linear regression (1530 × (1-e^-0.00306 g^) + 8.79), dashed line is linear regression (AUC = 4.55 g + 9.14). Points are means ± SEM.

The glycemic response curves elicited by WB0, WB5, WB10, WB20, WB35 and WB50 in the 10 subjects who tested all doses are shown in Figure 2A. The mean AUC for the different doses of white-bread, respectively, were 10.0 ± 2.2^a^, 37.5 ± 8.1^ab^, 67.2 ± 11.7^bc^, 117.3 ± 17.3^c^, 172.0 ± 27.3^d^, and 207.3 ± 31.4^d ^mmol × min/L (means with different letter superscripts differ significantly, p < 0.05). Over this range, the dose-response of AUC on dose of avCHO was well described by an exponential association model (Figure 2B). When the 60 mean AUC values for each subject at each dose were included in the regression analysis, the evidence in favor of the non-linear model (with random growth coefficient to account for subject effect) over the random-effects linear model was overwhelming (difference in corrected AIC of 30.8).

The mean AUC for all 48 subjects after WB0, WB5, WB10 and WB20 were 10.2 ± 1.3, 28.4 ± 2.4, 57.9 ± 4.6 and 99.5 ± 5.9 mmol × min/L, respectively, and each of these means was statistically significantly different from all of the others (least significant difference = 11.4) (Figure 2C). The average AUC for the 5–20 g doses of white bread was significantly increased by 16.5 ± 7.2 mmol × min/L (p = 0.027) by being female, by 1.01 ± 0.27 mmol × min/L (p = 0.0006) for every 1 year increase in age, and tended to be reduced by 8.7 ± 7.5 mmol × min/L (ns) by being Caucasian and to be reduced by 1.5 ± 0.9 mmol × min/L (ns) for every kg/m^2 ^increase in BMI. Over this range of avCHO, a linear regression model provided an almost equally good fit as the non-linear model (difference in corrected AIC of 2.5) (Figure 2D). Therefore, it was considered justified to use a linear model to calculate EGL.

The day-to-day variation of glycemic responses elicited by small doses of avCHO were determined in the 10 subjects who performed at least 2 tests of each of WB0, WB5, WB10 and WB20, the 9 who performed at least 3 tests, and the 9 who performed at least 4 tests of each of these doses. For each subject the within-subject coefficient of variation (CV = 100* SD/mean) of the AUC values for each dose of white bread was calculated. There was no significant effect of the number of repeats done on the average CV for any dose of bread. However, the average CV's decreased as the dose of bread increased, with the mean for WB0, 94%, being significantly greater than that for WB5, 65%, which, in turn, was significantly greater than those for WB10, 41%, and WB20, 31% (Table 1).

**Table 1:** Mean and within-individual variation of glycemic responses elicited by doses of white bread containing 0, 5, 10 and 20 g available carbohydrate.

		WB0	WB5	WB10	WB20
10 subjects with 2 – 4 repeated tests of each dose	n^1^	2.0	2.3	2.4	2.3
	AUC^2^	9.2 ± 6.1	21.7 ± 12.4	42.5 ± 16.7	87.9 ± 34.6
	CV^3^	85 ± 56%	73 ± 35%	48 ± 35%	35 ± 23%
					
9 subjects with 3 – 6 repeated tests of each dose	n	3.7	3.6	3.8	3.7
	AUC	11.8 ± 10.3	27.6 ± 14.9	49.0 ± 21.6	90.6 ± 27.5
	CV	103 ± 44%	59 ± 16%	44 ± 20%	30 ± 11%
					
9 subjects with 4 – 7 repeated tests of each dose	n	5.1	4.6	5.3	5.2
	AUC	8.1 ± 6.4	37.3 ± 22.6	65.7 ± 16.2	115.0 ± 27.3
	CV	95 ± 53%	62 ± 19%	30 ± 15%	26 ± 13%
					
All 28 Subjects	CV	94 ± 50%^a^	65 ± 25%^b^	41 ± 26%^c^	31 ± 16%^c^

^1 ^mean number of tests of each dose done by each subject.

^2 ^Mean ± SD incremental area under the glucose curve (mmol × min/L).

^3 ^Mean ± SD of within-subject coefficients of variation.

^abc ^Means with different letter superscripts differ significantly, p < 0.05.

### Estimation of GL from EGL

**Figure 3:**
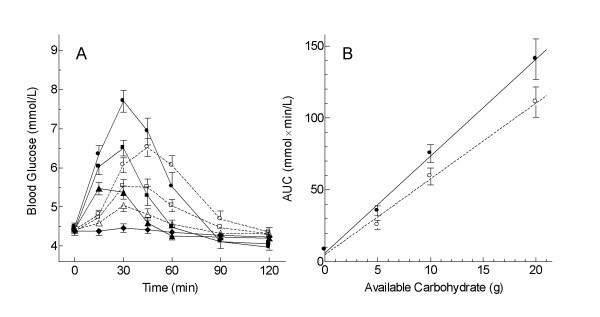
**Blood glucose responses elicited by various doses of oral glucose and white bread**. A: blood glucose concentrations after 0 (◆), 5 (▲), 10 (■), 20 (●) glucose and 5 (△), 10 (□) and 20 g (○) carbohydrate portions of white bread (each dose tested 2 times in 20 subjects); B linear regression of mean incremental areas under the curve (AUC) on grams carbohydrate (g) for glucose (●; AUC = 6.74 g + 6.0; r^2 ^= 0.997, p = 0.0014), and white bread (○; AUC = 5.28 g + 4.9; r^2 ^= 0.992, p = 0.0038). Points are means ± SEM.

The mean glycemic responses elicited by the different doses of white bread and glucose are shown in Figure 3A, with the mean AUC values shown in Table 2. There were highly significant effects of dose and subject for AUC after 5–20 g glucose (dose, p < 0.0001; subjects, p = 0.0001) and 5–20 g WB (dose, p < 0.0001; subjects, p < 0.0001). Among the 20 subjects, the mean AUC of the 5, 10 and 20 g carbohydrate doses of bread and glucose increased significantly with age, but was not significantly affected by BMI or sex. Being Caucasian was associated with a lower mean AUC which was not significant for glucose (difference of 13 ± 13 mmol × min/L) but was significant for WB (difference of 18 ± 9 mmol × min/L, p = 0.038). However, when the AUC after WB was expressed as a percentage of the AUC after the same dose of glucose, there was no significant effect of dose or subject. Similarly, the average EGL values for the 4 foods were not significantly related to sex, ethnicity, age or BMI.

**Table 2:** Glycemic responses, EGL and GL values for the 11 test meals in study 3.

Test Meal	AUC (mmol × min/L)	EGL (g)	GL* (g)	GL estimated from EGL**
Nothing (drink only)	8 ± 2	0.9 ± 0.2	0.6 ± 0.1	0.5
Chocolate Shake	8 ± 2	1.2 ± 0.3	0.7 ± 0.2	0.7
Chocolate Peanut Bar	21 ± 3	3.3 ± 0.6	2.3 ± 0.4	2.4
Caramel Nut Chew	25 ± 4	3.7 ± 0.6	2.7 ± 0.5	2.7
Pancake Mix	48 ± 7	7.1 ± 0.8	5.2 ± 0.5	5.3
White bread (5 g Av CHO)	26 ± 3	3.9 ± 0.4	2.7 ± 0.4	2.8
White bread (10 g Av CHO)	59 ± 6	10.3 ± 0.6	8.1 ± 0.7	7.8
White bread (20 g Av CHO)	111 ± 11	20.2 ± 0.2	16.0 ± 0.8	15.6
Glucose (5 g)	35 ± 3	5.7 ± 0.4	4.3 ± 0.3	4.2
Glucose (10 g)	75 ± 6	14.1 ± 0.9	10.7 ± 0.5	10.8
Glucose (20 g)	141 ± 14	26.4 ± 1.4	19.9 ± 0.2	20.4
Least Significant Difference	23	2.9	2.1	-

Values are means ± SEM.

* GL calculated from regression of AUC on dose of oral glucose using equation (7).

** GL estimated from EGL using equation (11).

The correlation coefficients of AUC on dose of carbohydrate from white bread for the 20 individual subjects ranged from r = 0.882 to r = 0.999 with a mean of 0.969 ± 0.008; this was not significantly different from the mean correlation coefficient for AUC on dose of glucose. The range of EGL values represented by the extremes of the 95% confidence band for the subject with an average correlation coefficient (Figure 1B) for each of the 4 foods (observed range in 20 subjects given in brackets) were: Chocolate Delight Shake, 0–7 g (0–4 g); Chocolate Peanut Bar, 0–9 g (0–8 g); Caramel Nut Chew, 0–10 g (0–8 g); Pancake Mix, 0–14 g (0–13 g). Thus, the observed range of variation of individual EGL values was similar to that expected from imprecision in the estimates of M and B.

The dose-response of AUC on grams avCHO was linear for glucose (r^2 ^= 0.997, p = 0.0014) and white bread (r^2 ^= 0.992, p = 0.0038) (Figure 3B). The y-intercepts were similar for bread, 4.9 ± 3.9, and glucose, 6.0 ± 2.9, but the slope for bread, 5.28 ± 0.33 mmol × min/L/g, was less than that for glucose, 6.74 ± 0.25 mmol × min/L/g (p = 0.024). Thus, for a given AUC, 5.28b + 4.9 = 6.74 g + 6.0, where b = grams of avCHO from white bread and g = grams of glucose. Solving this equation for g yields: g = (5.28b - 1.1)/6.74. Thus:

GL = 0.78 × EGL - 0.2     (11).

**Figure 4:**
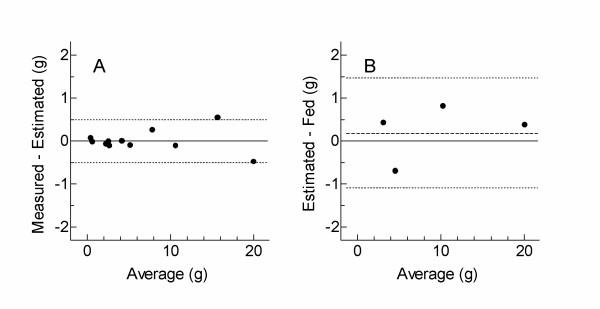
**Bland-Altman plots**. The dashed line represents the mean difference (on graph A, the difference = 0) and the dotted lines represent the 95% limits of agreement. A: comparison of measured and estimated GL values for 0, 5, 10 and 20 g carbohydrate from white bread and glucose and 4 low-carbohydrate foods. Measured values were calculated from the regressions of AUC on dose of glucose ingested for each subject; estimated values were derived from EGL. B: comparison GL values estimated from EGL for 3, 5, 10 and 20 g oral glucose and the amount of glucose fed. GL was estimated from EGL using equation (11) (see text) as follows: GL = 0.78 × EGL - 0.2.

The EGL and GL values for the different test meals are shown in Table 2. Bland-Altman analysis showed that the difference between GL values calculated from the regression of AUC on dose of oral glucose and GL estimated from EGL was 0.0 g with 95% limits of agreement of ± 0.5 g (Figure 4A). Thus equation (11) is accurate to within 0.5 g carbohydrate.

### Accuracy of EGL

When the EGL values for 3 g glucose were subjected to 2-way ANOVA, there was no significant difference between subjects (p = 0.55). The mean EGL value was 4.65 ± 0.63, which equals an GL value of 3.4 g. Bland-Altman analysis showed that the average difference between GL estimated from EGL and the amount of glucose actually consumed for the 3, 5, 10 and 20 g doses of oral glucose was -0.2 ± 0.3 g, with the 95% confidence interval of the differences being 1.1 to -1.5 g (Figure 4B).

## Discussion

The results show that the method described here for measuring EGL of low carbohydrate foods is accurate to within ~1 g carbohydrate and at least sensitive enough to detect a glycemic load as small as 3 g glucose. In addition, it was shown that as little as 5 g avCHO from white bread raises blood glucose by a detectable amount. However, within-subject variability of glycemic responses elicited by small doses of carbohydrate relative to the absolute response (i.e. the signal:noise ratio) is high. The dose-response curve of AUC on dose of avCHO was nearly linear over the range of 0–20 g avCHO, but significantly non-linear over the range 0–50 g avCHO.

With only 10 subjects, the minimum increment in avCHO intake associated with a statistically significant difference in glycemic response was 10 g, but, with all 48 subjects, the mean glycemic responses elicited by 0, 5, 10 and 20 g avCHO differed statistically from each other. However, it should be noted that most subjects repeated the tests of the different doses of white-bread more than once, with some of them repeating the tests more than 4 times each; this would improve the precision of the estimate of each subject's response to each dose. Thus, a significant difference in glycemic response elicited by a 5 g increment in avCHO intake may not be able to be detected with 50 subjects if each subject tests each dose only once.

Within-subject variability can be expressed either in absolute or relative terms. SD represents the absolute variation of glycemic responses, and the mean within-individual SD of the AUC values tended to increase from 6–10 mmol × min/L to 27–35 mmol × min/L as the dose of carbohydrate increased from 0 to 20 g. However, when expressed as CV, within-individual variation decreased from 93% to 65%, 41% and 31% for the 0, 5, 10 and 20 g carbohydrate doses of white-bread, respectively. In absolute terms, the magnitude of within-subject variation of glycemic responses elicited by small doses of carbohydrate is small, but relative to the average response, i.e. the signal:noise ratio, the variation is very large, with the "noise" (2 × SD) being larger than the "signal" for 5 g carbohydrate and 80% of the "signal" for 10 g carbohydrate. The implication of this is that the precision of the estimate of the glycemic response of a food, relative to that elicited by a reference carbohydrate, would be low with doses of avCHO <20 g. The mean within-individual CV for the 20 g avCHO dose of white bread, 31%, is similar to that for 50 g avCHO portions of white bread or glucose, which, for normal subjects, has been reported to be in the range of 19–29% [17,21].

The shape of the dose-response curve of AUC on dose avCHO was non-linear for doses between 0 and 50 g. The exponential model used for the non-linear regression was of the form AUC = A × (1-e^-Bg^) + C where g is the grams of avCHO, A is a constant describing the maximum AUC attained, C is the y-intercept and B is the rate constant which describes the rate at which the curve reaches its maximum value – i.e. the degree of curvature of the line. We previously found that this model explained 94% of the variation of mean glycemic responses of 0, 25, 50 and 100 g avCHO portions of 4 different starchy foods [18]. The value of the parameter A depends, in part, upon the glucose tolerance status of the subjects being tested. However, within a single group of subjects, the value of the parameter A was directly proportional to the GI of the food, while the value of parameter B was the same for different foods [18].

Glycemic load (GL) is a measure of the extent to which a given amount of food raises blood glucose, which each unit of GL being equal to the blood glucose raising potential of 1 g glucose. GL is defined as GI × g, where g is the amount of avCHO in grams, however, the present results suggest that GI × g is not an accurate way of determining GL. For low carbohydrate foods GI × g cannot be used to determine GL because GI cannot be measured accurately. GI is defined as the AUC elicited by the food expressed as a % of that after a dose of glucose containing the same amount of avCHO; it has been suggested that the dose of avCHO should be at least 25 g [22]. The present results support this suggestion because with doses of avCHO <20 g the within-individual variation of AUC values is very large relative to the mean which would result in a very imprecise estimate of GI. It has been suggested that GL could be measured directly as F × 50/G where F is the glycemic response elicited by the food and G is the glycemic response elicited by 50 g glucose [23]. However, this would overestimate GL for low carbohydrate foods because of the non-linear nature of the dose response curve of AUC on grams avCHO.

The present results also show that GI × g is also not an accurate way of determining the GL of high-carbohydrate foods, because GI × g increases linearly with g, whereas the actual dose-response curve is non-linear for doses of avCHO over 20 g. Recently Venn et al. [14] assessed the validity of GL calculated as GI × g by measuring the GL of various doses of high carbohydrate foods directly using a method analogous to that used here, except that a non-linear dose-response curve was used because doses of avCHO up to 78 g were used. Venn et al. [13] concluded that, for practical purposes, GI × g provides a good estimate of GL. However, the difference between GL and GI × g was directly related to the grams avCHO (r = 0.73, p = 0.002) in the portion of food tested and GI × g significantly overestimated GL for doses of food containing >25 g avCHO by about 8–10%. This is consistent with the present results. Thus, we believe that GL should not be calculated as GI × g but needs to be measured using an approach analogous to that used here. Across the range of 0–20 g avCHO a linear equation can be used to calculate EGL of GL, but for EGL values >20 g, use of a non-linear regression equation is required for an accurate result.

In the present study we verified the accuracy of EGL by showing that when the EGL values of 4 small doses of glucose (3, 5, 10 and 20 g) was used to estimate GL, the average difference between GL and the amount actually consumed was 0.2 g with all differences being less than 1 g. Although the difference between estimated GL and the amount of glucose consumed did not appear to be related to the dose of glucose across the range of 3 g to 20 g, only 4 doses were used which may not be enough to detect such a relationship.

The sensitivity of EGL could be considered to be the smallest value which can be detected as being significantly different from zero. This value is less than the minimum increment in carbohydrate intake associated with a significant difference in glycemic response, because the latter involves a statistical comparison of 2 independently variable values. We did not set out here to determine the sensitivity of EGL. However, when the EGL value for 3 g glucose was determined in 10 subjects on 2 occasions, both values were very significantly >0, and 19 of the 20 individual values were >0. This suggests that the method is at least sensitive enough to detect the glycemic response elicited by 3 g glucose.

## Conclusion

We conclude that the method of determining EGL described here is a valid measure of the blood glucose raising potential of low carbohydrate foods containing <20 g available carbohydrate or having an EGL value <20 g. Across this range, EGL is accurate to within about ± 1 g glucose, and is at least sensitive enough to detect a glycemic response equivalent to that produced by 3 g glucose with 10 subjects.

## Competing interests

Thomas Wolever is president and part owner of Glycemic Index Laboratories, Inc. (GI Labs), a contract research organization which performed the studies reported here, and Glycaemic Index Testing, Inc. a corporation which provides technical services to laboratories and is involved in education and promotion of the glycemic index. The article processing charge for this manuscript was paid for by Dr. Wolever. Alison Gibbs received consulting fees from GI Labs. Matthew Spolar is Vice President of Atkins Nutritionals, Inc. Elinor Hitchner was an emloyee of Atkins Nutritionals, Inc. Colette Heimowitz is an employee of Atkins Nutritionals, Inc.

## Authors' contributions

TW assisted in the design of the experiment, was responsible for collection of the data, performed the primary analysis of the data and wrote the manuscript. AG provided statistical advice, assisted in analysis of the data and reviewed the manuscript. MS and EH assisted in the design of the experiment and reviewed the manuscript. CH reviewed the manuscript.
